# Low Microfilaremia Levels in Three Districts in Coastal Ghana with at Least 16 Years of Mass Drug Administration and Persistent Transmission of Lymphatic Filariasis

**DOI:** 10.3390/tropicalmed3040105

**Published:** 2018-09-26

**Authors:** Dziedzom K. de Souza, Joseph Otchere, Collins S. Ahorlu, Susan Adu-Amankwah, Irene A. Larbi, Edward Dumashie, Frances A. McCarthy, Sandra A. King, Samson Otoo, Dickson Osabutey, Joseph H. N. Osei, Kojo M. Sedzro, Odame Asiedu, Samuel K. Dadzie, Irene Ayi, Benjamin Marfo, Nana-Kwadwo Biritwum, Daniel A. Boakye

**Affiliations:** 1 Department of Parasitology, Noguchi Memorial Institute for Medical Research, College of Health Sciences, University of Ghana, Legon-Accra, Ghana; jotchere@noguchi.ug.edu.gh (J.O.); edumashie@noguchi.ug.edu.gh (E.D.); ababafua@gmail.com (F.A.M.); sking@noguchi.ug.edu.gh (S.A.K.); sotoo@noguchi.ug.edu.gh (S.O.); osabutey@noguchi.ug.edu.gh (D.O.); josei@noguchi.ug.edu.gh (J.H.N.O.); sdadzie@noguchi.ug.edu.gh (S.K.D.); iayi@noguchi.ug.edu.gh (I.A.); dboakye@noguchi.ug.edu.gh (D.A.B.); 2 Department of Epidemiology, Noguchi Memorial Institute for Medical Research, College of Health Sciences, University of Ghana, Legon-Accra, Ghana; cahorlu@noguchi.ug.edu.gh (C.S.A.); sadu-amankwah@noguchi.ug.edu.gh (S.A.-A.); ialarbi@noguchi.ug.edu.gh (I.A.L.); sedzro@gmail.com (K.M.S.); 3 Neglected Tropical Diseases Programme, Ghana Health Service, Accra, Ghana; odame_114@yahoo.com (O.A.); marfobenjamin2002@yahoo.com (B.M.); 4 Bill and Melinda Gates Foundation, Seattle, WA 98109, USA; nkadbiritwum@gmail.com

**Keywords:** *Wuchereria bancrofti*, elephantiasis, lymphatic filariasis, transmission, control, Ghana

## Abstract

Ghana has been implementing mass drug administration (MDA) of ivermectin and albendazole for the elimination of lymphatic filariasis (LF) since the year 2000, as part of the Global Programme to Eliminate Lymphatic Filariasis (GPELF). It was estimated that 5–6 years of treatment would be sufficient to eliminate the disease. Tremendous progress has been made over the years, and treatment has stopped in many disease endemic districts. However, despite the successful implementation of MDA, there are districts with persistent transmission. In this study we assessed the epidemiology of LF in three adjoining districts that have received at least 16 years of MDA. The assessments were undertaken one year after the last MDA. 1234 adults and 182 children below the age of 10 years were assessed. The overall prevalence of circulating filarial antigen in the study participants was 8.3% (95% CI: 6.9–9.9), with an estimated microfilaria prevalence of 1.2%. The microfilarial intensity in positive individuals ranged from 1 to 57 microfilariae/mL of blood. Higher antigen prevalence was detected in males (13.0%; 95% CI: 10.3–16.2) compared to females (5.5%; 95% CI: 4.1–7.2). The presence of infection was also highest in individuals involved in outdoor commercial activities, with the risks of infection being four- to five-fold higher among farmers, fishermen, drivers and artisans, compared to all other occupations. Using bednets or participating in MDA did not significantly influence the risk of infection. No children below the age of 10 years were found with infection. Detection of Wb123 antibodies for current infections indicated a prevalence of 14.4% (95% CI: 8.1–23.0) in antigen-positive individuals above 10 years of age. No antibodies were detected in children 10 years or below. Assessment of infection within the *An. gambiae* vectors of LF indicated an infection rate of 0.9% (95% CI: 0.3–2.1) and infectivity rate of 0.5% (95% CI: 0.1–1.6). These results indicate low-level transmission within the districts, and suggest that it will require targeted interventions in order to eliminate the infection.

## 1. Introduction

Lymphatic filariasis (LF) is a common parasitic disease of major public health importance in tropical and subtropical countries, which are economically less endowed. About 90% of all lymphatic filariasis is caused by *Wuchereria bancrofti*, one of the three parasitic filarial nematodes [1]. These parasites are transmitted through the bite of infective mosquitoes of various genera. In highly endemic areas the disease may result in the disfiguring of the limbs and enlargement of the scrotum (hydrocele).

Owing to the availability of tools, the Global Programme to Eliminate Lymphatic filariasis (GPELF) was launched in 2000, with the aim of eliminating the disease as a public health problem by the year 2020 [2]. Albendazole (ALB) in combination with ivermectin (IVM) or diethylcarbamazine (DEC) were the main drugs available for the large-scale control of LF [3]. However, a combination of all three drugs—ivermectin + diethylcarbamazine + albendazole (IDA)—was recently approved for use in settings where LF is not co-endemic with onchocerciasis or loiasis [4]. The treatment strategy relies on the assumption that if the microfilaria (mf) reservoir in the human host is reduced below a certain threshold, transmission of *W. bancrofti* by anopheline vectors could be interrupted [5]. By this strategy it was estimated that five to six rounds of mass drug administration (MDA) will be required to eliminate the disease. Ghana is one of the first countries to have started the implementation of the GPELF in 2000 [6]. Being co-endemic for onchocerciasis, the recommended treatment regimen is IVM + ALB given once a year. These drugs mostly target mf in the blood, with limited macrofilaricidal and sterilization effect on the adult worms [7]. Recent studies, however, suggest that administering ALB twice a year has macrofilaricidal activity [8]. In Ghana, despite several years of treatment activities, districts with persistent transmission have been identified [9,10], necessitating the search for alternative and improved strategies for the control of LF. Alternative and effective treatment regimens and strategies have been recommended in order to achieve the LF elimination goals, such as, treatment to be given at shorter intervals or at increased dosage [3,7,11]. Thus, a cluster-randomized study was initiated in 2017, with the aim of assessing the impact of twice-yearly treatment with IVM + ALB in communities with persistent transmission in Ghana [12]. Following the baseline parasitological surveys, the data was analyzed to better understand the epidemiology of LF in the study areas. The information from this baseline analysis will help to implement targeted interventions, including adequate community sensitization required to achieve maximum impact, as well as monitoring the effects of the interventions.

## 2. Materials and Methods

### 2.1. Ethical Approval and Consent

Approval for the study was received from the Ghana Health Service Ethics Review Committee (GHS-ERC: 04112/2016) and the NMIMR IRB (CPN 062/16-17) with Federal Wide Assurance Registration (FWA 00001824). Community consent was sought for the study at community durbars, during which the aims of the study, procedures, risks, and benefits were explained to community leaders and members. Written informed consent was received from all study participants. For participants below the age of 18 years parental consent was sought, with written assent from children 12–17 years.

### 2.2. Study Design

The study was undertaken as part of a cluster randomized survey previously described [12]. The baseline parasitological study was conducted in selected LF endemic communities with a history of at least 16 years of MDA. Following community entry, study participants were recruited and enrolled into the study. Each participant was interviewed through a questionnaire, administered to obtain data on age, sex, occupation, place of residence, use of treated bednet, and participation in MDA. Each participant also provided daytime finger-prick blood, which was tested for *W. bancrofti* antigen by the rapid method. Antigen-positive individuals were followed up for night-blood samples, for the identification of mf by microscopy and detection of *W. bancrofti* L3 larval stage antigen-specific IgG4 antibodies by ELISA. Filter paper blood spots were obtained from all child participants aged 5–10 years old and tested for ELISA. Mosquitoes were also collected from some of the communities and dissected to ascertain infection/infectivity with *W. bancrofti*.

### 2.3. Study Sites and Participants

The study was conducted in 18 LF endemic villages in the Ahanta West, Nzema East and Ellembele Districts in the Western Region of Ghana. The Ahanta West and Ellembele districts share borders with the Nzema East district, and have the same ecology. The main occupations in the districts are farming and fishing. The Nzema East and Ellembele districts started MDA in 2002 while the Ahanta West district started in 2001. Details of the study districts, communities and sample size calculation were previously described [12].

Study sites were selected following a review of the Neglected Tropical Disease Programme (NTDP) sentinel and spot check site monitoring data, as well as recommendation from the District Health Management Team (DHMT). The sample size determination took into consideration the null hypothesis that an additional MDA is not more effective than the standard single dose per annum treatment, and the alternate hypothesis that an additional MDA is more effective than the standard single dose. With prevalence between sites ranging from 1% to 18%, the sample size was determined assuming an effect size of 0.4, power of 0.80, 37% non-response rate (determined from a previous study). Thus, 80 participants were targeted from each community, with a total of 1440 participants for the entire study.

### 2.4. Demographic Data Collection

A computer-assisted personal interviewing (CAPI) using Census and Survey Processing System (CSPro) was employed to obtain data on age, sex, occupation, place of residence, use of treated bednet, and participation in MDA.

### 2.5. Blood Sample Collection and Analyses

Daytime finger-prick blood was collected from all study participants and tested for *W. bancrofti* antigen using the Filaria Test Strip (FTS) [13]. Filter paper blood spots were prepared for all participating children 5–10 years old.

Two milliliters of night-blood was collected from each *W. bancrofti* antigen-positive individuals into EDTA tubes for the identification of mf using the nucleopore filtration method [14]. Briefly, 1 mL of blood was transferred into a test tube containing 9 mL of 3% acetic acid, and the mixture filtered through a 25 mm diameter Nucleopore Track-Etch filter membrane with pore size 5 µm. The membrane was placed with the top down on a clean glass slide, and examined under a light microscope for the identification of mf. The intensity of infection was estimated as mf per mL (mf/mL) of blood.

All collected filter paper blood spots from children and night-blood samples were tested for the detection of IgG4 antibodies specific for *W. bancrofti* L3 larval stage antigen [15], using the Wb123 ELISA and following the manufacturer’s protocol [16].

### 2.6. Mosquito Collection and Dissection for W. bancrofti

Mosquitoes were collected in six of the study communities, where antigen-positive individuals were identified. Two communities were selected in each study district. In each community, ten houses were randomly selected and mosquitoes were collected from houses, using the pyrethrum spray method [17]. In each community, the collection was done in the morning, from 5 a.m. to 8 a.m. The collected mosquitoes were identified using morphological identification keys [18], and subsequently dissected for identification of *W. bancrofti* larval stages. Briefly, mosquitoes were individually placed on a labeled glass slide, separated into head, thorax, and abdomen, and gently teased apart in a drop of saline solution. The dissected parts were observed under the microscope for any *W. bancrofti* larval stages (L_1_–L_3_).

### 2.7. Data Analysis

The data was combined for all districts since they shared borders and were similar in terms of ecology, occupation, and language. The data collected was analyzed using STATA (Version 15) and MedCalc Software (Version 18.6). Prevalences were estimated as percentages, with 95% confidence intervals. The chi-square test was used in the comparison of proportions. Statistical significance was estimated at *p* value ≤ 0.05.

## 3. Results

### 3.1. Demographic Characteristics of the Populations

1462 individuals consented to participate in the study. Out of these, 560 (38.3%) were males (median: 29 years; mean: 31.11 years; SD ± 19.62) and 902 (61.7%) females (median: 34 years; mean: 35.66 years; SD ± 19.54). The age range of study participants was 5–99 years, with a median of 32 years and a mean of 33.9 years (SD ± 19.7). The occupations with the highest proportion of the study participants were farming (26.7%) and schooling (26.5%), followed by trading (12.6%) and fishing (12.4%). Working as a health professional was the least proportion (0.6%) whilst 4.7% were unemployed (Table 1).

The majority of study participants 889/1462 (60.81%) reported staying in the community all their life. Of the participants who did not live in the community all their life, 9/573 (1.57%) had been in the community for less than 1 year while the remaining 564/573 (98.43%) had been in the community for more than 1 year. 1020/1462 (68.8%) participants reported owning a mosquito net and 753/1462 (51.5%) reported sleeping under a mosquito net. 109/196 (55.6%) of children 10 years or below reported sleeping under a mosquito net. 1293/1462 (88.44%) participants reported ever participating in the MDA, while 169/1462 (11.56%) never took part in the MDA. Table 2 presents the distribution of study participants and the number of years they have participated in MDA. 31.3% of participants could not recall the number of times they participated in the MDA. 56.9% took part in the MDA five times or less.

### 3.2. Wuchereria bancrofti Antigen Prevalence by FTS

**Figure 1:**
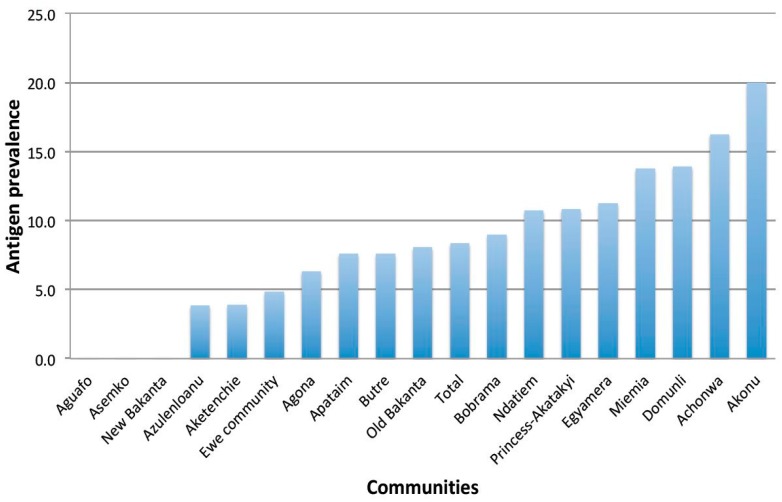
Prevalence of *W. bancrofti* antigen among study participants as categorized by communities.

Overall, there was antigen prevalence of 8.3% (95% CI: 6.9–9.9; *N* = 1418) in the study communities. The prevalence in Ahanta West, Ellembele and Nzema East districts was 10.6% (95% CI: 8.0–13.8), 4.5% (95% CI: 2.8–6.7) and 9.9% (95% CI: 7.4–13.0) respectively. The highest *W. bancrofti* antigen prevalence was recorded in Akonu (20.0%) followed by Achonwa (16.3%), Miemia (13.8%) and Domunli (13.9%). The prevalence in remaining villages ranged from 0 to 11.3% (Figure 1).

**Figure 2:**
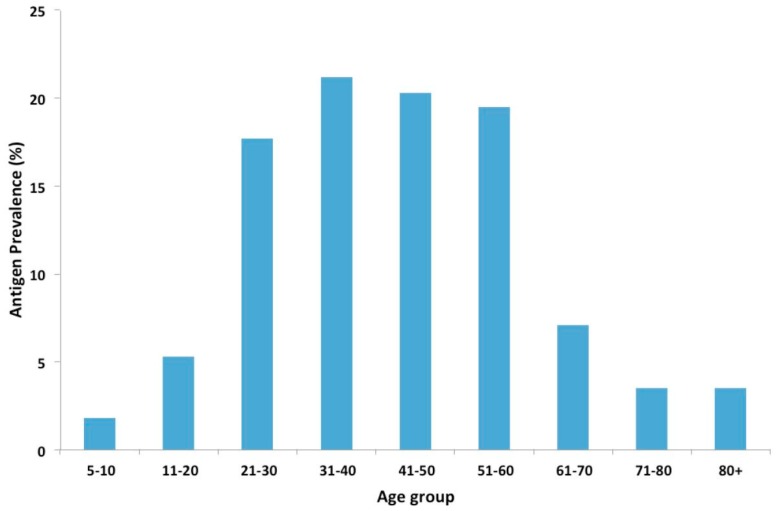
Prevalence of *W. bancrofti* antigen among participants by age groups.

Overall, 70/538 (13.0%; 95% CI: 10.3–16.2) males and 48/878 (5.5%; 95% CI: 4.1–7.2) females were positive for *W. bancrofti* antigen. Distribution of antigen-positive participants among age groups revealed the majority of infections in the 21 to 60 years categories (Figure 2). Only two participants (1.8%) in the 5–10 years category were identified with *W. bancrofti* antigen, and they were both 10 years of age. There were statistically significant differences in the levels of infection between gender (χ^2^ = 24.1; *p* < 0.0001).

Bivariate analysis of antigenemia with bednet usage revealed that 9.4% (95% CI: 7.2–12.0) of those who did not use bednets were positive for antigen, with 8.5% (95% CI: 6.6–10.7) of the users also being positive. In terms of participation in MDA, 5.0% (95% CI: 2.2–9.6) of respondents who indicated that they did not participate in the most current MDA were positive for antigen whilst 8.8% (95% CI: 7.3–10.5) of respondents who took part in MDA were found to be positive for antigen. No statistically significant relationship was found between antigen positivity and the use of bednet and participation in MDA (Table 3). There was however a significant association with occupation. For the four occupations with the highest infection prevalence, the risks of infection significantly increased nearly four- to five-fold, compared to all other occupations (Table 4). Of these four occupations, 58.3% (*N* = 662) of individuals involved were females and 41.7% were males. Univariate and multivariate logistic regression indicated that gender and age are important factors in determining the presence or absence of the infection (Table 5). Females were 47% less likely to be infected than males. Also, the odds of being infected increases by 2% as a person advances in age.

### 3.3. Prevalence and Intensity of Microfilaria

Ninety-seven (97) of 117 antigen-positive individuals were available for night-blood collection. Of these only 14 tested positive for mf. The mean intensity was 11 mf/mL of blood, with a range of 1–57 mf/mL of blood. Table 6 presents the prevalence and intensity of mf in the study districts. No significant differences (*p* = 0.55) were observed in the mf prevalence between males and females.

Mf prevalence = $\left(\frac{b}{a}\right)$ × $\left(\frac{d}{c}\right)$ × 100, where *a* = the number of individuals in the community examined for CFA, *b* = number of those examined for CFA being positive, *c* = number of CFA positives examined for mf, and *d* = number of those examined for mf being positive.

### 3.4. Prevalence of W. bancrofti IgG4 Antibodies

Blood samples from 279 participants composed of 97 antigen-positive individuals (above 10 years of age) and 182 children (aged 5 to 10 years) were subjected to Wb123 ELISA test to detect the presence of IgG4 antibodies specific to the third larval stage of *W. bancrofti*. The antibody prevalence among antigen positive individuals above 10 years was 14.4%. No antibodies were detected in children 5–10 years (Table 7).

### 3.5. W. bancrofti Infection and Infectivity Rates in An. gambiae

The *An. gambiae sensu lato* collected from selected communities were dissected and observed under the microscope for *W. bancrofti* larval stages (L_1_, L_2_ and L_3_). The results (Table 8) indicate an overall infection (presence of any stage of parasite) rate of 0.9% (95% CI: 0.3–2.1). Infection was only observed in Achonwa (2.2%) and Ndatiem (0.8%). The overall infectivity (presence of L_3_ only) rate was 0.5% (95% CI: 0.1–1.6).

## 4. Discussion

This paper presents the baseline epidemiological situation of LF in 18 communities in three endemic districts in Ghana that have received 16 to 18 years of MDA without interrupting transmission, prior to randomization and assignment to treatment groups for the evaluation of a twice-yearly treatment regimen with IVM + ALB. In this study, 1462 participants consented and provided demographic information, of which 1418 provided day blood samples for antigen testing using the FTS. The overall antigen prevalence in all the study districts was 8.3%, which is far above the minimum 2% level, below which transmission could be said to have been interrupted [20,21]. Similarly, the district level antigen prevalences were also higher than the recommended level.

The mf levels, on the other hand, were lower than expected. The overall mf prevalence was 1.2%, which was just slightly higher than the 1% level recommended for the interruption of transmission [20,21]. However, except for the mf prevalence in Ahanta West that was above the threshold (1.6%), both Nzema East and Ellembele had lower mf prevalence of 0.5% and 0.8%, respectively. While the mf prevalence might qualify Nzema East and Ellembele to proceed to carry out transmission assessment surveys (TAS) in children [20], the levels of antigen prevalence do not. The high antigen prevalence could be due to residual adult antigen from resolved infections, with studies reporting the circulation of antigen in the blood for up to three years after treatment [22].

In this study, antigen prevalence increased with age, with similar trends reported in other studies [23,24]. This increase in antigen prevalence with age can be attributed to the increasing exposure of the population to *W. bancrofti* infection. Higher antigen prevalence was also observed in males compared to females. This is consistent with studies, which showed a lower mean prevalence of infection in females than in males [25,26]. Higher prevalence with increased risks of infection was also observed in people involved in outdoor commercial activities such as farmers, fishermen, and drivers. Earlier studies in Ghana attributed infections to the occupational activities [27,28], with activities such as farming and fishing exposing individuals to mosquito bites [29,30]. Individuals involved in outdoor activities, and especially males, may therefore represent high-risk groups that could be targeted during MDAs, in order to reduce the prevalence of infection in LF endemic areas.

The presence of antigen-positive but mf-negative individuals could be due to very low levels of mf circulating in the blood or infections where adults worms do not produce mf [31]. This could be a result of the sterilization effect of albendazole on adult worms [8,32], especially with repeated treatment.

The assessment of Wb123 antibodies for the detection of early exposure to infection [15] indicated that 14.4% of antigen-positive adults were infected. Incidentally, antibodies were only detected in individuals found with mf. Given that antibodies may persist in the blood stream for years, it is impossible to determine if the presence of antibodies in individuals with mf is due to recent infections. The mf prevalence observed could be due to low compliance to MDA as only 8.6% of the population reported receiving at least five rounds of MDA. Since IVM and ALB, administered once a year, have mainly microfilaricidal effects with limited macrofilaricidal effect, any lapse in treatment will result in a large number of mf circulating in the blood, thus perpetuating the transmission cycle. As a result of the low compliance, adult worms in infected individuals may continue to produce mf, leading to the high prevalence observed among antigen-positive individuals.

The use of the Wb123 as a new tool for assessment in post-MDA monitoring is promising [15,33]. While none of the children below the age of 10 years were found infected, it is important to note that the study was not designed to assess infection in children, as undertaken during transmission assessment surveys. Further studies and guidelines to implementation for the Wb123 will be required [34].

The generally low mf levels observed in the study population raise questions about the importance of low-level transmission, especially in communities that have received several years of treatment. The importance of vector-parasite interaction therefore comes to play. In our study communities, the main LF vectors belong to the *Anopheles gambiae* complex, with *An. melas* being a major vector [35,36]. *Mansonia* species have also been reported in the districts, with both *An. melas* and *Mansonia* spp. reported to exhibit the vector-parasite process of limitation [37,38], thus facilitating disease transmission at low mf levels in the community. The dissection of *An. gambiae* collected from some of the study communities revealed the presence of L_3_ infective stage larvae, an indication of active transmission. The exophilic nature of the vector species in the study communities also implies that transmission may occur outdoors, and may explain why individuals involved in outdoor commercial activities are at higher risks of infection. Despite these findings, there are limitations to the mosquito infection data as these did not take into consideration a sample size and the power required to make meaningful deductions. From the results, it can be observed that *W. bancrofti* larvae were not identified in communities where low mosquito numbers were collected, implying that larger sample sizes will be required in such communities. However, collecting a large enough mosquito sample size would have required significant financial inputs, which unfortunately was not possible due to the funding available for the study. Nonetheless, the collection of mosquitoes in all communities was based on the same methods in the same month. As such, the numbers of mosquitoes collected could reflect the transmission potential in the communities.

This study provides information on the persistent LF transmission in three districts of Ghana that have received at least 16 years of MDA. Earlier studies in selected communities in the study areas indicated that mf load and antigen prevalence in infected individuals decreases after treatment and gradually increases within six months [10]. Thus, administering ivermectin and albendazole to infected individuals twice in the year may help accelerate LF elimination in these settings [10]. However, compliance to MDA was found to be low. Qualitative assessments indicated that while community members were aware of the purpose of the MDA, factors such as personal attitudes, the health system, absence of overt disease, social structure, and fear of adverse effects of drugs affected the compliance to the MDA [39]. Considering the importance of low-level transmission in these settings, it may be useful to target interventions at high-risk individuals such as males, farmers, and fishermen in order to increase compliance in individuals most likely to serve as reservoirs of infection.

This study also emphasizes the need for comprehensive assessments in communities with persistent transmission, using a combination of assessment tools in human and vector populations. The parasitological, immunological and xenomonitoring assessments all revealed the presence of infection and transmission of the disease. Taken together, the results indicate the need for a thorough epidemiological understanding in communities with persistent LF transmission, in order to plan appropriate/tailored control interventions and achieve the LF elimination goals.

## Figures and Tables

**Table 1:** Occupational distribution of study participants.

Occupation	Frequency (%)
Health professional	9 (0.6)
Teaching	19 (1.3)
Driving	23 (1.6)
Retired/aged	38 (2.6)
Unemployed	69 (4.7)
Artisanship	80 (5.5)
Fishing	181 (12.4)
Trading	184 (12.6)
Students	387 (26.5)
Farming	390 (26.7)
Others	80 (5.5)

**Table 2:** Distribution of participants by number of years in mass drug administration (MDA) participation.

Years Participation	No. of Participants (%)
Do not know	451 (31.34)
1	164 (11.40)
2	217 (15.08)
3	195 (13.55)
4	118 (8.20)
5	124 (8.62)
6	35 (2.43)
7	21 (1.46)
8	20 (1.39)
9	14 (0.97)
10	40 (2.78)
11	1 (0.07)
12	2 (0.14)
13	-
14	1 (0.07)
15	8 (0.56)
16	26 (1.81)
17+	2 (0.14)

**Table 3:** Bivariate analysis of factors associated with lymphatic filariasis. ** *p* < 0.01.

	LF Outcome			
	Positive (%)	Negative (%)	$\chi^{2}$	*p*-Value
**Bednet Use**				
No	56 (9.4)	542 (90.6)	0.33	0.56
Yes	62 (8.5)	671 (91.5)		
**MDA Treatment**				
No	8 (5.0)	153 (95.0)	2.68	0.10
Yes	110 (8.8)	1145 (91.2)		
**Occupation**				
Artisanship	9 (13.8)	56 (86.2)	55.09	0.001 **
Driving	4 (19.0)	17 (81.0)		
Farming	54 (14.2)	326 (85.8)		
Fishing	23 (13.1)	153 (86.9)		
Hairdressing	1 (7.7)	12 (92.3)		
Health profession	0 (0)	8 (100)		
Retired from working	2 (5.6)	34 (94.4)		
Studying	7 (1.9)	369 (98.1)		
Teaching	2 (10.5)	17 (89.5)		
Trading	8 (4.6)	167 (95.4)		
Unemployed	5 (7.4)	63 (92.6)		
Other	3 (3.8)	76 (96.2)		

**Table 4:** Relative risk of the four main occupations with highest infection prevalence compared to all other occupations.

Occupation	Relative Risk	95% CI	*p*-Value
Artisanship	3.8	1.9–7.8	0.0002
Driving	5.3	2.0–13.7	0.0006
Farming	3.9	2.5–6.1	<0.0001
Fishing	3.6	2.1–6.1	<0.0001

**Table 5:** Logistic regression estimates of the risk factors of LF infection.

	Univariate		Multivariate	
Factor	OR (95 CI)	*p*-Value	OR (95 CI)	*p*-Value
Age	1.02 (1.01–1.02)	0.000	1.02 (0.01–1.03)	0.000
Gender	0.53 (0.38–0.73)	0.000	0.47 (0.34–0.66)	0.000
Using a mosquito net	1.24 (0.90–1.73)	0.187	1.23 (0.89–1.72)	0.216
Occupation	1.00 (0.99–1.00)	0.321	1.00 (0.99–1.00)	0.158
Time in community	0.84 (0.61–1.17)	0.303	0.95 (0.68–1.33)	0.765
Participation in MDA	1.26 (0.75–2.24)	0.410	1.14 (0.67–2.07)	0.643
District	0.99 (0.81–1.20)	0.891	1.06 (0.39–2.86)	0.904
Village	1.00 (0.98–1.02)	0.844	1.00 (0.91–1.10)	0.976

**Table 6:** Prevalence of microfilaria and mean intensity of infection among study participants by district.

District	No. Tested	No. +ve for mf	Mf Prevalence in % *	95% CI	Range of Mf Intensity (mf/mL)
Ahanta West	41	8	19.5 (1.6)	8.8–34.9	1–43
Nzema East	43	5	11.6 (0.5)	3.9–25.1	1–57
Ellembelle	13	1	7.7 (0.8)	0.2–36.0	2
**Total**	**97**	**14**	**14.4 (1.2)**	**8.1–23.0**	**1–57**

* Values in () estimated based on the Simonsen’s formula [19] with.

**Table 7:** Prevalence of *W. bancrofti* IgG4 antibodies by age group.

*W. bancrofti* IgG4 Antibodies Detection Outcome			
Age Range (Years)	Negative *n* (%)	Positive *n* (%)	95% CI
5–10	182 (100)	0 (0.0)	0.0–2.0
>10 *	83 (85.6)	14 (14.4)	8.1–23.0

* For those above 10 years, IgG4 antibodies were assessed only in antigen-positive individuals.

**Table 8:** *W. bancrofti* infection and infectivity rates in *An. gambiae* in selected study communities.

Communities	No. Analyzed	No. with Any Stage Larvae (L_1_, L_2_, L_3_)	Infection Rate (95% CI)	No. with L_3_ Larvae Only	Infectivity Rate (95% CI)
Achonwa	138	3	2.2 (0.5–6.2)	2	1.5 (0.2–5.1)
Akatekyi	66	0	0.0 (0.0–5.4)	0	0.0 (0.0–5.4)
Domunli	20	0	0.0 (0.0–16.8)	0	0.0 (0.0–16.8)
Old Bakanta	30	0	0.0 (0.0–11.6)	0	0.0 (0.0–11.6)
Bobrama	43	0	0.0 (0.0–8.2)	0	0.0 (0.0–8.2)
Ndatiem	254	2	0.8 (0.1–2.8)	1	0.4 (0.01–2.2)
**Total**	**551**	**5**	**0.9 (0.3–2.1)**	**3**	**0.5 (0.1–1.6)**
